# Improved Surface-Enhanced-Raman Scattering Sensitivity Using Si Nanowires/Silver Nanostructures by a Single Step Metal-Assisted Chemical Etching

**DOI:** 10.3390/nano11071760

**Published:** 2021-07-06

**Authors:** Ioannis Kochylas, Spiros Gardelis, Vlassis Likodimos, Konstantinos P. Giannakopoulos, Polycarpos Falaras, Androula G. Nassiopoulou

**Affiliations:** 1 Section of Condensed Matter Physics, Department of Physics, National and Kapodistrian University of Athens, Panepistimiopolis Zografou, 15784 Athens, Greece; ikochyla@phys.uoa.gr (I.K.); vlikodimos@phys.uoa.gr (V.L.); 2 Institute of Nanoscience and Nanotechnology, National Center for Scientific Research “Demokritos”, Agia Paraskevi, 15341 Athens, Greece; k.giannakopoulos@inn.demokritos.gr (K.P.G.); p.falaras@inn.demokritos.gr (P.F.)

**Keywords:** silicon nanowires, metal-assisted chemical etching, surface enhanced Raman spectroscopy, Ag dendrites, Ag aggregates

## Abstract

In this study, we developed highly sensitive substrates for Surface-Enhanced-Raman-Scattering (SERS) spectroscopy, consisting of silicon nanowires (SiNWs) decorated by silver nanostructures using single-step Metal Assisted Chemical Etching (MACE). One-step MACE was performed on p-type Si substrates by immersion in AgNO_3_/HF aqueous solutions resulting in the formation of SiNWs decorated by either silver aggregates or dendrites. Specifically, dendrites were formed during SiNWs’ growth in the etchant solution, whereas aggregates were grown after the removal of the dendrites from the SiNWs in HNO_3_ aqueous solution and subsequent re-immersion of the specimens in a AgNO_3_/HF aqueous solution by adjusting the growth time to achieve the desired density of silver nanostructures. The dendrites had much larger height than the aggregates. R6G was used as analyte to test the SERS activity of the substrates prepared by the two fabrication processes. The silver aggregates showed a considerably lower limit of detection (LOD) for SERS down to a R6G concentration of 10^−13^ M, and much better uniformity in terms of detection in comparison with the silver dendritic structures. Enhancement factors in the range 10^5^–10^10^ were calculated, demonstrating very high SERS sensitivities for analytic applications.

## 1. Introduction

Surface enhanced Raman scattering (SERS) has been proved to be a powerful method for analyzing chemical and biological species down to single molecules in solutions or at surfaces [1,2,3,4,5]. Combination of SERS with the continuous miniaturization of Raman instruments, which became portable and cheaper, could provide an excellent, alternative analytical tool to well-established techniques such as chromatography and mass spectroscopy, for various applications ranging from drug detection [6] to food safety [7], which can benefit from fast and accurate, on the spot, chemical analysis.

The sensitivity of conventional Raman scattering is inherently low due to the fact that only a small fraction of photons is used for detection (inelastically scattered photons) leading to very small cross sections of the order of 10–30 cm^2^/sr [8]. This intrinsic limitation can be overcome by the use of rough and/or nanostructured substrates covered by coinage metals, mainly silver and/or gold nanoparticles, or copper, which provide significant enhancement factors of the order of 10^4^–10^7^ as a result of the excitation of localized surface plasmons [9,10,11]. SERS can take place near a metallic nanoparticle, which is irradiated at the resonance frequency or near resonance of its localized surface plasmons resulting in a large enhancement of both the local and radiation fields in the vicinity of the metallic nanoparticles and thus in a marked amplification of the Raman signal from adsorbed molecules [12]. This electromagnetic field enhancement can be most prominent at hot spots on a SERS substrate, as the electromagnetic field near plasmonic nanostructures is generally non-uniform. Such hot spots can be nanogaps between metallic nanoparticles, nanotips, or nanogaps between the substrate and the metallic particle [12]. In particular, single molecule SERS detection becomes effective when analyte molecules are located at interparticle junctions of the metallic nanoparticles [13].

Intensive research efforts have been accordingly devoted to improve the sensitivity of SERS substrates, and nowadays, there are reports of electromagnetic enhancement factors (EFs) of the order of 10^8^ when averaged over the whole substrate or of the order of 10^10^ on hot spots [14], whereas there are some early reports of even higher EFs regarding single molecule detection [15,16]. More recently, an EF of the order of 10^14^ was reported for a patterned superhydrophilic-superhydrophobic SERS substrate, where the analyte was concentrated locally on superhydrophilic sites surrounded by syperhydrophobic regions enabling SERS detection with high sensitivity [17].

One of the most established approaches to achieve higher SERS sensitivity compared to two-dimensional (2D) SERS substrates is the three-dimensional (3D) configuration of metal nanostructures and particularly silver and gold ones. Such 3D nanostructures offer a higher surface-to- volume ratio, i.e., larger specific surface area and thus higher sensitivity. In addition, these structures show EFs higher by more than one order of magnitude compared to the 2D planar metal SERS substrates, due to the increase of hot spots’ density in a 3D structure. For example, SERS substrates consisting of a dense array of silicon nanowires (SiNWs) fabricated by metal-assisted chemical etching (MACE) of silicon substrates and coated by silver nanoparticles have shown enhancement factors of the order of 10^8^ [18].

In another approach SERS substrates with silicon nanorods covered by gold nanoparticles were fabricated by placing polystyrene spheres in a close-packed configuration on a silicon substrate followed by reactive ion etching of the polystyrene spheres and deposition of a gold layer. Metal-assisted chemical etching of silicon and removal of gold and polystyrene spheres resulted in an ordered array of silicon nanopillars, which after decoration by gold nanoparticles led to EF above 10^7^ [19]. A similar top-down approach of an ordered array of silicon nanorods covered by silver nanoparticles showed EFs of the order of 10^6^ [20]. Most recently, SERS substrates consisting of a dense network of silver dendrites were developed on silicon substrates by MACE. Specifically, the silver dendrite network was developed by chemical etching of silicon in a solution of AgNO_3_ with HF/H_2_O and applied as SERS substrate for the sensitive detection of lysozyme with EF of 2.4 × 10^6^ [21].

In this study, we fabricated SiNWs by MACE and decorated them with silver nanoparticles in order to explore their potential as substrates for sensitive molecule detection by SERS. The use of SiNWs aims at increasing the reactive surface area by the 3-dimensional morphology. Specifically, we explored two different methods of growing the silver nanostructures on the SiNWs substrates. In the first method, we fabricated SiNWs by MACE and removed any silver nanoparticles, which were formed during SiNWs growth by immersion in HNO_3−_. The SiNWs free of any silver deposits were re-immersed in the initial solution used for the MACE growth for a few minutes. Depending on the immersion time, silver aggregates were formed mainly at the SiNWs tips and only few silver nanoparticles managed to penetrate and be deposited on the SiNWs walls. We explored two subcases using this method. Initially, we fabricated SiNWs about 6 μm long. We found that it was very difficult to cover the SiNWs walls with silver nanoparticles and most of them were deposited at the SiNWs tips in the form of aggregates. We switched to the fabrication of much shorter SiNWs of lengths of about 200–300 nm. In this case, the SERS sensitivity increased considerably mainly due to the much less void space among the SiNWs, as the nanowires were much wider providing a larger surface to accommodate the silver aggregates. In the second method, we retained the idea of growing short SiNWs, however, without removing the silver nanostructures grown during the MACE process. This single-step MACE process resulted in the formation of silver dendrites at the SiNWs tips with considerably larger heights than those of the aggregates formed by the first method. In both cases, a 3D structure was produced. Comparative evaluation of the Ag-SiNWs substrate SERS performance produced by the two processes was performed by using Rhodamine R6G as a prototype analyte under 514 nm laser excitation. Diffused reflectance and transmittance measurements performed on the SERS substrates showed broad spectral plasmonic excitation-related features at wavelengths in the vicinity of the 514 nm laser line. This ensures plasmonic excitation of the silver nanostructures, which can cause electric field enhancement of both incident photons of the laser and of the re-emitted Raman ones. SERS mapping demonstrated that the SERS substrates with the silver dendritic structures rendered similar or slightly larger SERS signals than those with the silver aggregates. However, the substrates with the silver aggregates demonstrated spectacular uniformity over their whole surface at R6G concentrations down to 10^−7^ M. At R6G concentrations as low as 10^−13^ M, only the SERS substrates with the silver aggregates still demonstrated SERS signals with the characteristic peaks of R6G however only at certain hot-spot locations. Elsewhere, the R6G SERS signal was suppressed by a background SERS signal similar to that observed on the SERS substrates without the R6G analyte. This was more evident in the SERS substrates with the silver dendritic structures where the background SERS signal was present even at R6G concentration of 10^−9^ M. EFs in the range 10^5^–10^10^ were calculated.

## 2. Materials and Methods

The SERS substrates were fabricated by the MACE method, developed in our labs originally for the fabrication of devices for solar cell applications and metal-insulator-semiconductor (MIS) capacitors [22,23]. A p-type (100) oriented monocrystalline Si wafer was used to template the formation of the silver nanostructures by a single-step MACE. SiNWs were grown by immersion of different pieces from the p-type Si wafer into an aqueous solution of AgNO_3_ mixed with hydrofluoric acid (HF). Two different methods were used to grow the SERS substrates. In the first method, we selected etching times of 5 and 30 min, producing samples containing shorter (about 300 nm long) SiNWs and longer (about 6 μm long) SiNWs, respectively. In this method, the as-formed SiNWs were dipped in an aqueous solution of HNO_3_ for 4 min, in order to dissolve the silver nanostructures that were formed on the SiNWs during MACE. Then, the clean SiNWs from any silver residuals were re-immersed in a fresh MACE solution with the same composition as that used for the silicon etching process for 3 and 6 s in order to decorate the SiNWs with silver nanoparticles. For the longer SiNWs, we also used a re-immersion time of 10 s. In the second method, different etching times of 2, 3.5 and 5 min were applied, leading to the formation of SiNWs with length of 200–300 nm. In this method, the as-grown silver nanostructures that were grown during SiNWs formation in the MACE solution were not removed. Table 1 summarizes the fabrication conditions of the SERS substrates and the corresponding names. We evaluated the performance of the SERS substrates produced by the different methods in terms of the SERS limit of detection (LOD) and enhancement factor (EF) for different concentrations of R6G used as analyte to test the samples.

The morphology of the SERS substrates was investigated by scanning electron microscopy (SEM). Diffused reflectance measurements were carried out in the range between 200 and 1000 nm by a Cary 60 UV–VIS (Agilent, Santa Clara, CA, USA) spectrophotometer equipped with a fiber optic diffuse reflectance accessory. For transmittance measurements, a special preparation process of the samples was followed [24]. According to this process, an adhesive coating of polyvinyl acetate (PVAc) was deposited on glass substrate by spin-coating and heated at 80 °C. The MACE prepared SiNWs sample with the silver dendritic structure facing down was glued on the glass substrate. After cooling down to room temperature, a lateral force was applied to the SiNWs sample by tweezers resulting in the detachment of the SiNWs with the silver dendritic structure from the substrate. The transmittance measurements were carried out in the range between 200 and 1000 nm using a spectrometer LR1 by ASEQ instruments coupled with suitable optical fibers. SERS measurements were carried out by micro-Raman spectroscopy using a Renishaw inVia Reflex microscope with a solid-state laser emitting at 514 nm. The laser beam was focused onto the samples by means of a ×50 (ΝA = 0.75) objective lens on a Leica DMLC microscope down to a spot size of about 1 μm. The power density of the laser was low and smaller than 0.06 mW/μm^2^ to avoid local heating effects. To evaluate the performance of the SERS substrates, the R6G dye in aqueous solutions was used as model analyte in various concentrations. A 50 μL drop of each of these solutions was casted on the different SERS substrates and allowed to get adsorbed and dried and then measured under identical conditions with one accumulation for 10 s exposure time.

## 3. Results and Discussion

### 3.1. Structure and Morphology

**Figure 1:**
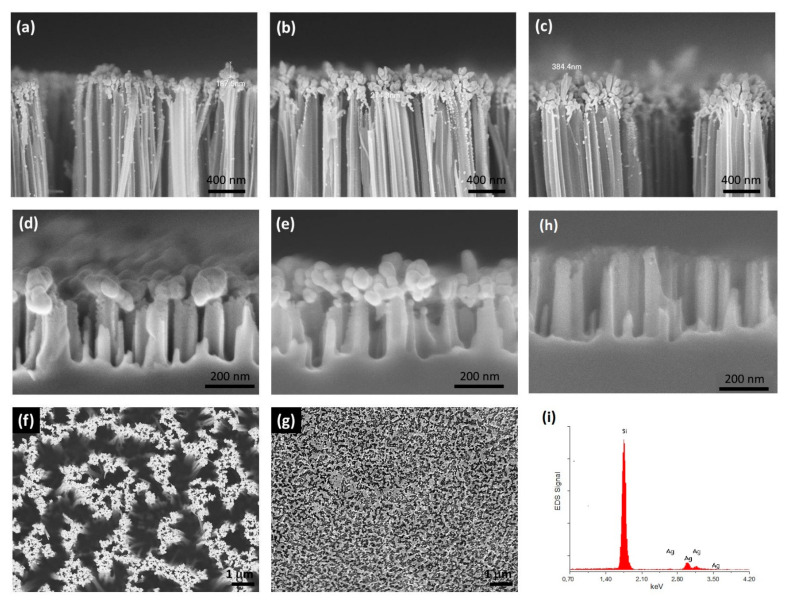
SEM cross-section images of (**a**) A30M3S, (**b**) A30M6S, (**c**) A30M10S, (**d**) A5M3S and (**e**) A5M6S. Silver aggregates grow mainly at the SiNWs tips, and only a few silver nanoparticles may decorate the nanowire walls for the longer SiNWs. The aggregates increase in size with increasing re-immersion time in the MACE solution. Top view SEM images showing the top surface of (**f**) A30M3S, (**g**) A5M3S, (**h**) SEM image after removing the Ag aggregates from A5M3S, (**i**) EDS spectrum obtained from an area of (**g**).

Figure 1 shows cross-sections of the longer SiNWs grown for etching time of 30 min using the first method. Specifically, Figure 1a–c depicts the A30M3S, A30M6S and A30M10S samples, respectively. The SiNWs had lengths of about 6 μm. Most of the silver nanostructures were grown at the SiNWs tips in the form of aggregates with larger sizes as the re-immersion time in the MACE solution increased. Only few silver nanoparticles can be viewed on the walls of the SiNWs, indicating limited penetration along the nanowires. Figure 1d,e shows the corresponding cross-sections for the shorter SiNWs samples, A5M3S and A5M6S, respectively, grown for etching time of 5 min using the first method. In that case, the SiNWs were about 300–350 nm long. The silver nanostructures exclusively formed at the tips of the SiNWs in the form of aggregates with larger sizes as the re-immersion time in the MACE solution increased without any indication of deposition at the walls.

Figure 1f,g compares the top surfaces of the SERS samples with the longer SiNWs and those with the shorter ones fabricated by the first method, i.e., A30M3S and A5M3S, respectively. It is evident that the longer SiNWs SERS samples provide much more empty space among the SiNWs. Figure 1h shows a SEM image of A5M3S after removing the Ag aggregates. Only the SiNWs are left. Figure 1i shows an Electron Dispersive Spectroscopy (EDS) spectrum obtained from the top surface of A5M3S (Figure 1g) showing only Ag and Si.

**Figure 2:**
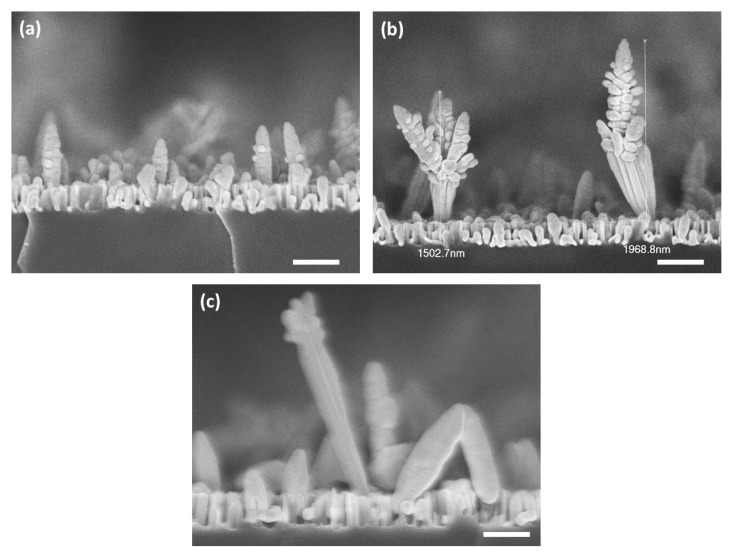
SEM cross-section images of (**a**) B2M, (**b**) B3.5M and (**c**) B5M samples. The dendritic silver nanostructures are grown between the SiNWs and increase in height and size with increasing etching time.

Figure 2a–c depicts cross-sections of the SERS substrates B2M, B3.5M and B5M, respectively. The SiNWs in these samples have lengths between 250–350 nm depending on the etching time. The silver nanostructures show an upward development, are elongated and present a dendritic configuration. These structures are higher as the etching time increases. Specifically, for the sample that was etched for 2 min, the silver nanostructures heights are less than 1 μm. For the sample etched for 3.5 min, silver nanostructures of about 2 μm high can be observed whereas for the sample that was etched for 5 min, silver nanostructures of more than 2 μm high can be observed. In this method, the silver dendritic structures were grown between the SiNWs, whereas in the former method, silver aggregates were grown at the tips of the SiNWs. The reason for this difference is that silver nanostructures start to grow at the active silver nanoparticle/Si interface, where the Si etching takes place. Silver nanoparticles formed due to AgNO_3_ reduction precipitate randomly at the Si surface. At these precipitation sites, Si etching initiates and evolves vertically forming the SiNWs. Thus, in the first method, where the silver nanoparticles are grown during the few second post-immersion of the bare SiNWs samples, the silver nanostructures are formed at the tips of the SiNWs, as it is most probable for Si etching to start at these sites. Due to the short immersion time, the silver nanostructures do not have the time to evolve into elongated dendritic-like structures, in contrast to those grown by the second method, and eventually are formed as aggregates at the SiNWs tips, as shown in Figure 1.

### 3.2. Optical Properties

**Figure 3:**
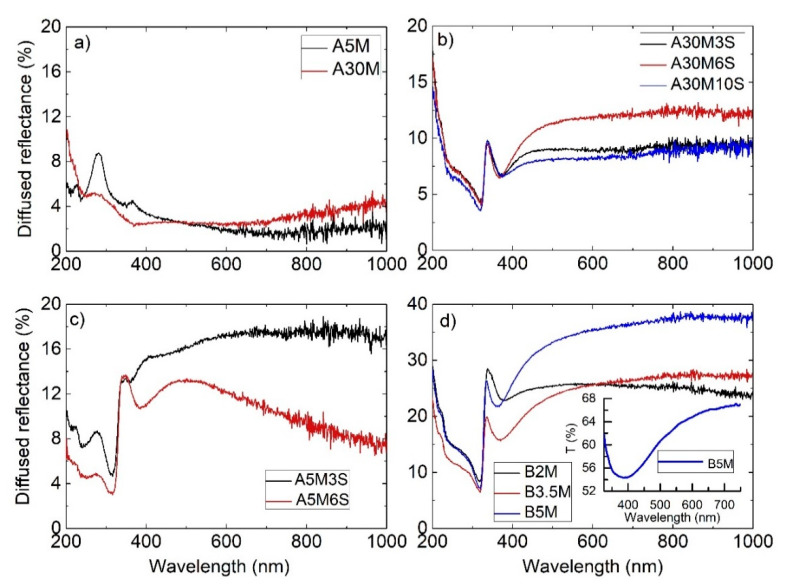
Diffused reflectance spectra of the (**a**) bare SiNWs A5M and A30M; (**b**) long Ag-SiWNs A30M3S, A30M6S and A30M10S; (**c**) short Ag-SiWNs A5M3S and A5M6S and (**d**) B2M, B3.5M and B5M samples fabricated by the second method. The inset in (**d**) shows the transmittance spectrum through the silver dendritic nanostructure that was removed from the surface of the B5M sample.

Diffused reflectance (DR%) spectra were obtained from both the bare SiNWs that were cleaned of any silver residues and from the silver-decorated SiNWs. Figure 3a displays the DR% spectra of the bare SiNW A5M and A30M samples grown by MACE for 5 and 30 min, respectively, from which silver residues were removed by post-treatment in HNO_3_. For the 5-min etched sample that consists of short SiNWs, the characteristic reflectance peaks of bulk Si at 280 and 365 nm can be clearly distinguished [25]. Figure 3b,c shows the corresponding DR% spectra for the longer (A30M3S, A30M6S and A30M10S) and shorter (A5M3S and A5M6S) Ag-SiWNs, while Figure 3d displays the corresponding spectra for the B2M, B3.5M and B5M SERS samples prepared by the second method.

Besides the higher reflectance observed for all the Ag-SiNWs SERS samples, a distinctive difference in the shape of their spectra can be observed in comparison with those of the bare SiNWs. Specifically, a dip between 338 and 600 nm with a minimum at about 370 nm can be observed in all SiNWs SERS substrates without significant modifications between the different samples. This feature can be related to the plasmonic excitation of the silver nanostructures, in perfect agreement with those reported for porous Si decorated by silver nanostructures after immersion in aqueous AgNO_3_ solution [26].

Comparing the DR% spectra of all SERS samples, it is evident that the most reflective samples, showing also the most intense dip features related to plasmonic excitation, are the B-series ones with the long silver dendritic nanostructures. The plasmonic excitation-related dip in the DR% of these samples is more intense than that in the A-series, where smaller silver aggregates were formed. Comparing the DR% of the SERS samples within the A-series, the samples with the shorter SiNWs are the most reflective, since in these samples the SiNWs tips that are covered by the silver aggregates have a larger area, and the gaps between the SiNWs are much smaller (Figure 1).

To shed more light into the plasmonic excitation of the SERS SiNWs samples, transmittance measurements were carried out on the silver decorated SiNWs by transferring them on glass substrates using the method described before. The inset in Figure 3d shows the transmittance spectrum of sample B5M. A broad dip is observed around 385 nm similar to that observed in the DR% spectra, which can be attributed to the plasmonic excitation of the silver nanostructures. The dips in transmittance and diffused reflectance spectra are rather broad due to the large distribution in the sizes and the aspect ratios of the silver dendrites or aggregates [27]. It should be noted that the excitation wavelength of the 514 nm laser line used in the SERS experiments falls within the broad spectral width of plasmonic excitations offering the possibility of high sensitivity in SERS [28].

### 3.3. SERS Performance

#### 3.3.1. A and B Series SERS Substrates

**Figure 4:**
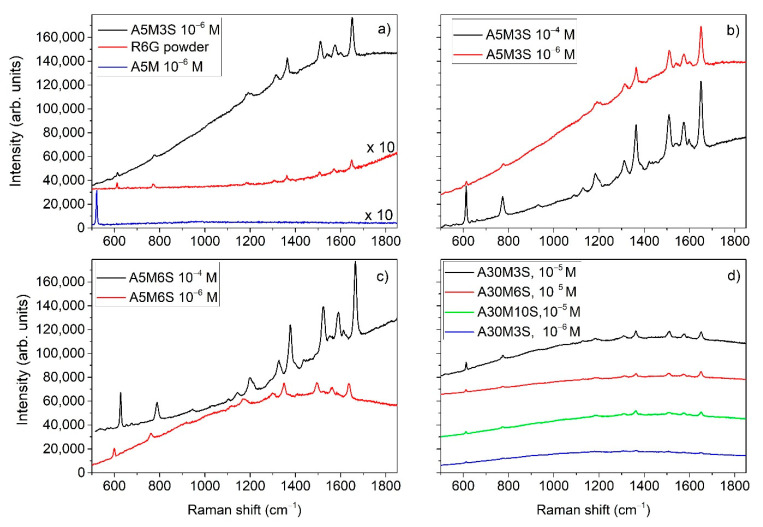
Raman spectra: (**a**) bare (A5M) and silver modified (A5M3S) SiNWs for 10^−6^ M R6G aqueous solution in comparison to R6G solid powder. SERS spectra of the A-series substrates (**b**) A5M3S, (**c**) A5M6S and (**d**) A30M3S for different R6G concentrations in the range of 10^−4^–10^−6^ M under identical conditions at 514 nm.

To evaluate the SERS performance of the SERS substrates investigated in this study, the R6G dye in aqueous solutions was used as analyte in various concentrations. A 50 μL drop of each of these solutions was casted on the different SERS substrates and allowed to get adsorbed and dried. Figure 4a compares the Raman spectra of bare (A5M) and silver modified (A5M3S) SiNWs for 10^−6^ M R6G aqueous solution in comparison to the reference spectrum of R6G solid powder, which is subsequently used to estimate the EF for the Ag-SiNWs substrates for different concentrations of R6G. The Raman spectrum of R6G is observed only after the decoration of the SiNWs with silver nanoparticles, whereas no signal can be detected if the R6G solution is casted on the bare SiNWs except for the Raman signal at 521 cm^−1^ originating from Si.

To identify the vibrational modes corresponding to these peaks, we relied on Reference [29], where a detailed comparison of the experimental frequencies with those theoretically predicted for the different vibrations of the R6G molecule is presented. We accordingly used the reported notation of Reference [29], where X refers to the xanthene ring vibration, A to the NHC_2_H_5_ group vibrations, P to the phenyl ring vibration with the COOC_2_H_5_ group, while M refers to the vibration of a pair of methyl group in the vicinity of the xanthene ring. Thus, the peak at 612 cm^−1^ originates from a combined motion of X and P. The 773 cm^−1^ corresponds to a combined motion of X, A and M. The 1128 cm^−1^ is a combined motion of X, A an P. The peaks at 1183 cm^−1^, 1362 cm^−1^, 1509 cm^−1^ and 1540 cm^−1^ are combined vibrational modes of X and A. The 1312 cm^−1^ is a combined motion of X, A and P. The peaks at 1575 cm^−1^, 1598 cm^−1^ correspond to vibrational motions of P. Finally, the most intense peak at 1650 cm^−1^ corresponds to vibrational motion of X. 

**Figure 5:**
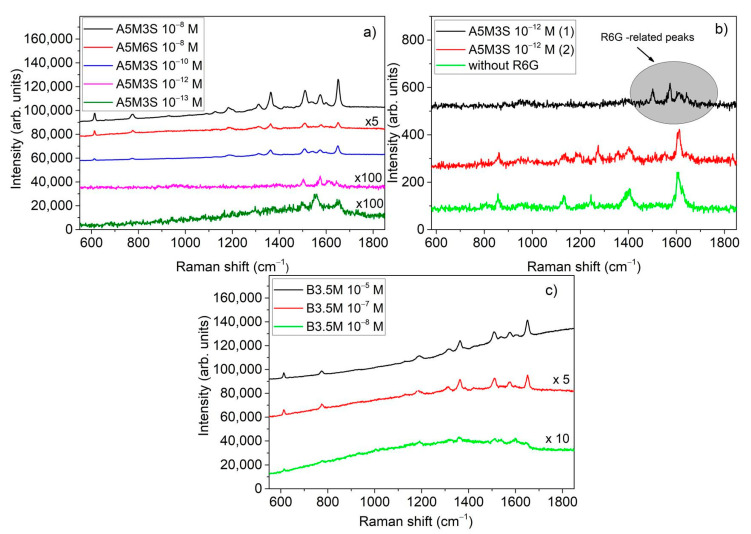
Raman spectra obtained from: (**a**) A5M3S and A5M6S for R6G concentrations in the range 10^−8^–10^−13^ M, (**b**) obtained from two different locations (1, 2) on A5M3S at R6G concentration of 10^−10^ M and compared with that obtained from a similar substrate without R6G, (**c**) B3.5M for RG6 concentrations in the range 10^−5^–10^−8^ M.

Figure 4b–d depicts SERS spectra of R6G obtained from A-series SERS substrates for concentrations up to 10^−6^ M. Figure 5a shows SERS spectra obtained from A-series SERS substrates for R6G concentrations ≤ 10^−8^ M. The lowest R6G concentration detected using substrates from A-series was 10^−13^ M. As can be deduced by Figure 4 and Figure 5, the A5M3S substrates demonstrate better sensitivity compared to that of A5M6S and A30M ones. Generally, at R6G concentrations lower than 10^−8^ Μ only certain hot-spots on the substrates demonstrated reliable SERS spectra which can be identified as Raman signal of R6G. Elsewhere, the SERS signal is suppressed by background peaks which can be observed even in specimens without R6G. An example of this reliability limitation in detection is depicted in Figure 5b, showing SERS signals obtained from two different locations on the substrate A5M3S at R6G concentration of 10^−12^ M compared with a SERS signal obtained from a similar substrate without R6G. SERS obtained from location (1) is the typical signal expected from R6G in contrast to that obtained from location (2) which differs and cannot be identified as SERS originated from R6G. The background Raman signal without R6G consisted of five peaks at 1608 cm^−1^, 1400 cm^−1^, 1244 cm^−1^, 1129 cm^−1^ and 860 cm^−1^ and was traced in both A- and B-series substrates. Although the exact origin of these spurious Raman peaks cannot be identified, such contamination issues have been encountered in SERS at the limit of single molecule detection, frequently including unwanted carbonaceous species that originate from the photodecomposition of organic species during Raman measurements [8,30]. In the case of B-substrates, this background signal was present at even higher R6G concentrations than in the case of A-substrates, reducing the limit of detection for R6G to 10^−8^ M. The reason for this is probably the considerably larger silver dendritic structures in comparison with the silver aggregates providing larger surfaces for such undesirable effects to occur.

From the SERS spectra obtained from the B-series SERS substrates, we choose to show here those obtained from B3.5M ones as they demonstrated the highest sensitivity within B-series. Figure 5c depicts SERS spectra obtained from these substrates for R6G concentrations 10^−5^–10^−8^ M. From the analysis of the Raman spectra above, it is evident that the most sensitive SERS substrates are the A5M3S ones which have silver aggregates. 

#### 3.3.2. Homogeneity of SERS Spectra over the Surface of A- and B- Series Substrates

**Figure 6:**
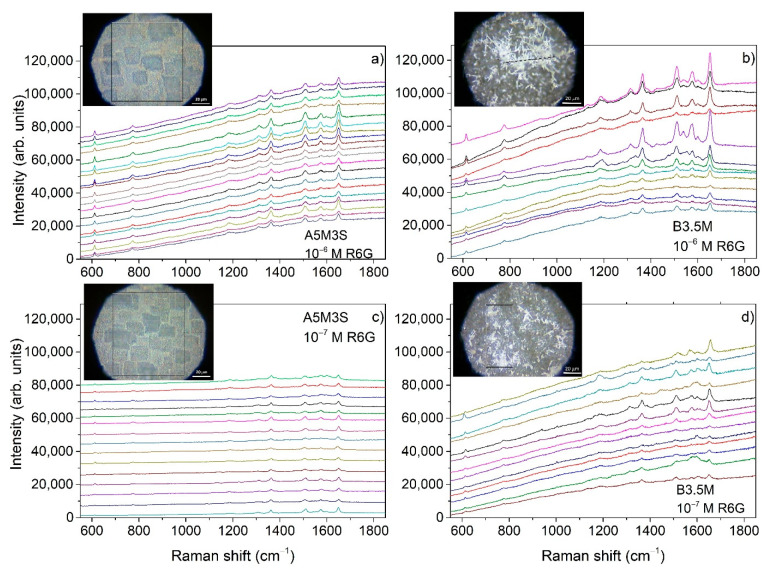
SERS mapping: (**a**,**b**) A5M3S and B3.5M, respectively, at R6G concentration of 10^−6^ M. (**c**,**d**) A5M3S and B3.5M, respectively, at R6G concentration of 10^−7^ M. The insets show images of the areas of the corresponding samples (squares or lines), magnified by the optical microscope of the RAMAN instrument, where mapping was performed. The scale bar is 20 μm.

To evaluate further the SERS performance of the A- and B-series substrates, we investigated the homogeneity of the SERS sensitivity over their surface. For this we carried out SERS mapping. Figure 6 shows SERS mapping for R6G concentrations of 10^−6^ M and 10^−7^ M. The mapping in the case of the A-series substrates has been performed over a larger area than in the case of B-series ones, as shown in the insets of Figure 6, due to the relatively different topography of the aggregates and dendrites in the two series of samples. Specifically, an area of 80 × 90 μm^2^ was mapped for the A-series sample. In the case of the B-series sample, a line of about 55 μm over the dendritic structure depicted in the corresponding image was mapped for the 10^−6^ M R6G concentration, and an area of 65 × 30 μm^2^ over the dendritic structure was scanned for the 10^−7^ M R6G concentration, as shown in Figure 6, where a group of random Raman spectra is displayed from each SERS map. These measurements were reproducible in different parts of the substrates as well as for different substrates fabricated under the same conditions.

Evidently, A-series substrates show remarkable homogeneity in the intensity of the R6G characteristic peaks in contrast to B-series which show larger intensity variation of the SERS signal with the location over the surface. The homogeneity of the SERS signal demonstrated in the A-series substrates is consistent with the homogeneity of the morphology of the silver aggregates in contrast with the highly inhomogeneous morphology of the silver dendrites as demonstrated by comparing the two morphologies in the insets of Figure 6 and in better resolution in the corresponding SEM images in Figure 1 and Figure 2.

In searching for the best SERS substrate made by the MACE method, it is evident from the SERS investigation carried out in this study that the best results are obtained by the method which produced the A-series samples. By reducing the etching time and producing shorter SiNWs, we improved considerably SERS detection. Therefore, within the A-series the shorter SiNWs showed the best results. This can be observed in Figure 4 by comparing the SERS signals, at R6G concentrations up to 10^−6^ M, obtained from samples A5M3S and A5M6S which have shorter SiNWs with those obtained from the sample A30M3S having longer SiNWs. One of the reasons is that the active area of the hot spots at the sample surface was increased considerably in comparison with that of the samples with the longer SiNWs where the etching time was 30 min. This is evident if we compare the SEM images showing the surface of the respective samples. In the case of the longer SiNWs samples, there is a considerable empty space as shown in Figure 1f, which reduces significantly the active area of the hot spots. From the A-series samples, the best SERS sensitivity is obtained from the A5M3S ones. Eventually, in the case of B-series SERS samples, retaining the short etching times and letting, during the etching process, the long silver dendritic structures grow in between and on the tips of the SiNWs without removing them, we would expect a considerable increase of the hot spots where SERS action can take place. Although up to a R6G concentration of 10^−8^ M we could detect clearly peaks of R6G in the SERS spectra (Figure 5a), above this concentration, we detected only background SERS signal consisting of peaks other than R6G similar to those depicted in Figure 5b for the case of SERS obtained from the silver-decorated samples without R6G. Additionally, the A5M3S samples showed remarkable homogeneity in SERS sensitivity compared to the B-series samples. Thus, the silver aggregates prove to be a better choice than the silver dendrites. 

#### 3.3.3. Enhancement Factors

To evaluate the nanostructures’ SERS performance, the enhancement factor (*EF*) was calculated both for the A-series and B-series SERS substrates for different R6G concentrations. For this calculation, we compared the SERS spectra obtained for the above-mentioned range of R6G concentrations with the conventional Raman spectrum obtained from the reference R6G powder sample performed under the same conditions. For this comparison, we have chosen two characteristic Raman peaks at 1509 cm^−1^ and 1650 cm^−1^. The formula to calculate *EF* is given by [21]:

(1) $$EF=\frac{I_{SERS}}{I_{RAMAN}}\times \frac{N_{RAMAN}}{N_{SERS}}$$

where $I_{SERS}$ is the intensity of a peak in the SERS spectrum and $I_{RAMAN}$ is the intensity of the same peak in the conventional Raman spectrum of the R6G refence powder sample. $N_{SERS}$ is the number of excited molecules within the area covered by the laser spot, $S_{spot}$, during SERS measurements. $N_{RAMAN}$ is the number of molecules contained in the volume *V* of the cylinder defined by the area $S_{spot}$ and h which is the depth of field penetration of the laser into the powder sample of R6G. Thus, $N_{RAMAN}$ is given by

(2) $$\;N_{RAMAN}=\frac{m}{M_{R6G}}N_{A}=\frac{\rho V}{M_{R6G}}N_{A}=\frac{\rho S_{spot}hN_{A}}{M_{R6G}}$$

where *m* is the mass of the R6G powder used for the Raman measurement, $N_{A}=6.022\times 10^{22}$ is the Avogadro number, $M_{R6G}=479.02\;g/\mathrm{mol}$ is the molar mass of R6G, $\rho =1.26\;g/{\mathrm{cm}}^{3}$.

$N_{SERS}$ is given by

(3) $$N_{SERS}=\frac{CVN_{A}S_{spot}}{S_{film}}$$

where *C* is the molarity of the R6G aqueous solution used in the SERS measurements, *V* = 50 μL is the volume of the drop casted on the SERS substrates, and $S_{film}=0.64\;{\mathrm{cm}}^{2}\;$ is the area of the SERS substrates. Eventually, the ratio $\frac{N_{RAMAN}}{N_{SERS}}$ is reduced to

(4) $$\frac{N_{RAMAN}}{N_{SERS}}=\frac{\rho S_{film}h}{CVM_{R6G}}$$

We calculated *h* using the following equation [27]:

(5) $$h=n\frac{\lambda }{Ν.A.^{2}}$$

where *n* is the refractive index of the R6G in solid form, which is 1.58 at 514 nm [31], *Ν.A.* = 0.75 is the numerical aperture of the x50 objective lens used in the experiments, and λ=514 nm is the laser wavelength, yielding the value of *h* = 1.44 μm.

In Table 2 and Table 3, we show examples of the calculated *EF* for the most SERS-sensitive substrates investigated in the present study for the Raman peaks at 1509 cm^−1^ and 1650 cm^−1^, respectively. 

From Table 2 and Table 3, it turns out that the A5M3S substrate shows the highest *EFs* among all the different SERS substrates investigated in the present study. One step MACE can be accordingly concluded to produce SERS substrates with limit of detection (LOD) detection for R6G down to 10^−13^ M, which is one of the best reported up to date among other studies using substrates prepared by similar methods [18,19,20,21], or comparable to other competitive methods combining super-hydrophobic surfaces with nanoplasmonic structures [17,32,33]. In addition, the calculated *EF*s in this study are among the highest reported in the literature [15,16,17].

It is well known that a marked improvement of SERS sensitivity takes place within the nano gaps between metal nanoparticles due to electric field enhancement there. Thus, aggregation of the metal nanoparticles can give rise to hot spots for SERS [34,35]. For example, it has been shown that aggregation of colloidal silver nanoparticles into flakes using salts can considerably improve SERS reproducibility as well as the limit of detection as aggregation provides the necessary hot spots [35]. The formation of silver aggregates at the tip of the SiNWs by MACE we presented here offers exactly this possibility of ample aggregation on the SERS substrates in a controllable surface density (as the surface area of of the SiNWs’ tips provides the scaffold for the growth of silver aggregates—longer and thinner SiNWs or shorter and wider SiNWs—depending on the MACE parameters) with remarkable uniformity and reproducibility in terms of morphology and SERS action. Although the SERS substrates with the silver dendrites show slightly higher SERS signals those with silver aggregates up to 10^−8^ M of R6G, they lag behind in terms of uniformity and also at R6G concentrations lower than 10^−8^ M suffer from limitations due to the spurious background signal.

## 4. Conclusions

In this work, 3D SERS substrates were developed by decorating SiNWs, fabricated by a single-step MACE process, with silver nanoparticles using two different routes. The first route produced silver aggregates at the tips of the SiNWs, while the second route produced silver dendrites at the tips of the SiNWs and among the SiNWs. The first method produced SERS substrates with considerably improved and much more uniform sensitivity over the surface than the second method. Specifically, the SERS substrates with the silver aggregates enabled SERS sensitivity for R6G detection (LOD) down to concentrations of 10^−13^ M which is a record value for this kind of SERS substrates. Additionally, enhancement factors of 10^10^ were estimated which are among the highest reported in the literature. Our purpose is to use these SERS substrates in order to develop optical biosensors for the immunochemical detection of cancer indices, proteins and indices of oxidative stress down to single molecule detection.

## Figures and Tables

**Table 1:** Specifications of SERS substrates.

SERS Substrates	First Method (A)	Second Method (B)	Etching Time (min, M)	Removal of Silver Residues	Re-Immersion Time (s, S)
A5M	YES	-	5	YES	-
A30M	YES	-	30	YES	-
A5M3S	YES	-	5	YES	3
A5M6S	YES	-	5	YES	6
A30M3S	YES	-	30	YES	3
A30M6S	YES	-	30	YES	6
A30M10S	YES	-	30	YES	10
B2M	-	YES	2	NO	-
B3.5M	-	YES	3.5	NO	-
B5M	-	YES	5	NO	-

**Table 2:** *EF* calculated for the different SERS samples at different R6G concentrations for the Raman peak at 1650 cm^−1^.

SERS Sample	10^−6^ M	10^−8^ M	10^−10^ M	10^−13^ M
A5M3S	2 × 10^5^	2 × 10^7^	7 × 10^8^	1 × 10^10^
A5M6S	10^5^	10^6^	-	-
B3.5M	10^5^	5 × 10^5^	-	-

**Table 3:** *EF* calculated for the different SERS samples at different R6G concentrations for the Raman peak at 1509 cm^−1^.

SERS Sample	10^−6^ M	10^−8^ M	10^−10^ M	10^−13^ M
A5M3S	2 × 10^5^	2 × 10^7^	5 × 10^8^	6 × 10^9^
A5M6S	10^5^	6 × 10^5^	-	-
B3.5M	10^5^	3 × 10^5^	-	-
